# Ga-68-Labeled Affibody Molecule-Based Radiopharmaceutical Targeting Platelet Derived Growth Factor Receptor Beta for Detection of Active Fibrosis in Patients with Myocardial Infarction

**DOI:** 10.3390/ph18121833

**Published:** 2025-12-01

**Authors:** Irina Velikyan, Karl-Henrik Grinnemo, Viktor Flodin, Stefan James, Ulrika Thelander, Michael Wagner, Sergey Rodin, Tanja Kero, Olle Korsgren, Olof Eriksson

**Affiliations:** 1 Science for Life Laboratory, Department of Medicinal Chemistry, Uppsala University, 751 83 Uppsala, Sweden; 2 Molecular Imaging and Medical Physics, Department of Surgical Sciences, Uppsala University, 751 83 Uppsala, Sweden; 3 Cardio-Thoracic Translational Medicine (CTTM) Laboratory, Department of Surgical Sciences, Uppsala University, 751 83 Uppsala, Swedenviktor.flodin@akademiska.se (V.F.); sergey.rodin@uu.se (S.R.); 4 Department of Medical Sciences, Uppsala University, 751 83 Uppsala, Sweden; 5 Department of Immunology, Genetics and Pathology, Uppsala University, 751 83 Uppsala, Swedenolle.korsgren@igp.uu.se (O.K.); 6 Antaros Tracer AB, 753 20 Uppsala, Sweden

**Keywords:** radiopharmaceutical, positron emission tomography (PET), Affibody molecule, myocardial infarction, fibrosis, preclinical and clinical studies, PDGFRβ, GMP, Gallium-68

## Abstract

**Introduction**: Platelet-derived growth factor receptor beta (PDGFRβ) is a key regulator of fibrogenesis. Non-invasive imaging of PDGFRβ expression may offer a novel approach to assess fibrotic remodeling, particularly in cardiac patients’ post-intervention, where fibrosis poses clinical risk. This study presents the GMP-compliant production of a novel PDGFRβ-targeted PET radiopharmaceutical, [^68^Ga]Ga-DOTA-Z09591 ([^68^Ga]Ga-ATH001), and its preclinical evaluation in mouse and human myocardial tissue, along with initial clinical imaging in patients with ST-elevation myocardial infarction (STEMI). **Methods**: The precursor was chemically synthesized and radiolabeled with gallium-68 using a fully automated, GMP-compatible system and a pharmaceutical-grade ^68^Ge/^68^Ga generator. Autoradiography, H&E, Sirius Red, Masson’s trichrome, and IHC staining were performed on infarcted mouse hearts and human myocardial biopsies. In vivo PET/MRI with [^68^Ga]Ga-ATH001, ^15^O-H_2_O, and gadolinium contrast was conducted in STEMI patients one week post-percutaneous coronary intervention. **Results**: [^68^Ga]Ga-ATH001 was produced with high radiochemical yield and purity. Autoradiography demonstrated specific, receptor-mediated binding of [^68^Ga]Ga-ATH001, co-localizing with PDGFRβ immunoreactivity, collagen deposition, and tissue damage. In STEMI patients, focal tracer uptake was observed in infarcted myocardium correlating with MRI-detected structural abnormalities and perfusion defects on ^15^O-H_2_O PET. Uptake in unaffected myocardium was low and homogeneous, consistent with minimal physiological PDGFRβ expression. **Conclusions**: [^68^Ga]Ga-ATH001 was successfully developed and validated for phase 0 clinical study. The tracer demonstrated PDGFRβ-specific binding in human fibrotic myocardium and enabled non-invasive detection of myocardial fibrogenic activity in STEMI patients. These findings support further clinical evaluation of [^68^Ga]Ga-ATH001 as a targeted molecular imaging agent for early assessment of post-infarction fibrosis.

## 1. Introduction

**Figure 1:**
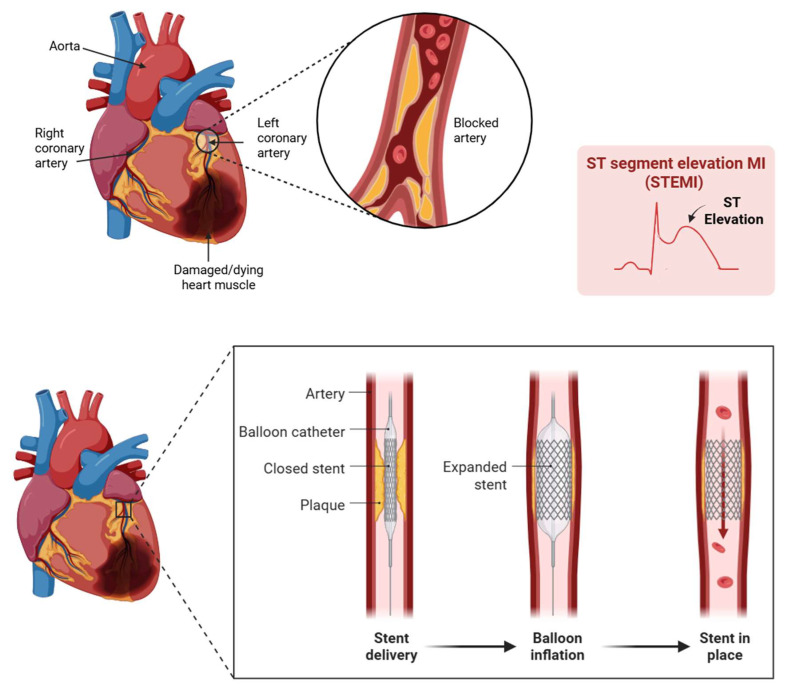
When a STEMI occurs, a major coronary artery is blocked, depriving heart tissue of oxygen. As seen here in the ST elevated myocardial infarction (STEMI)-gram, the blockage is clear. Immediate intervention through Percutaneous Coronary Intervention, or PCI, restores blood flow. Created with BioRender.com.

Myocardial infarction (MI) remains a leading cause of morbidity and mortality worldwide, often initiating a cascade of pathological processes that culminate in adverse cardiac remodeling [1]. Central to this remodeling is myocardial fibrosis, a hallmark of chronic injury that contributes to ventricular stiffening, impaired function, and eventual heart failure [2]. While timely revascularization with percutaneous coronary intervention (PCI) is critical, it paradoxically introduces myocardial ischemia–reperfusion (MIR) injury, which further contributes to fibrotic remodeling (Figure 1).

The extent of fibrosis, and thus the risk of post-infarction heart failure and mortality, is shaped not only by the time to reperfusion but also by the severity of MIR injury. Monitoring fibrotic activity is therefore essential: it enables early identification of patients at risk for adverse outcomes, guides therapeutic decisions, tracks response to intervention, and offers a valuable biomarker for understanding the healing process. By visualizing active fibrogenesis, clinicians and researchers can intervene more precisely and potentially improve long-term cardiac outcomes. However, current imaging modalities such as echocardiography, magnetic resonance imaging (MRI), and computed tomography (CT) detect structural or functional consequences of fibrosis but cannot capture the underlying biological activity during the early, potentially reversible stages of fibrotic remodeling [3]. Hence, there is an unmet need for a safe and non-invasive whole body accurate and quantitative imaging of fibrogenesis. Molecular imaging with positron emission tomography (PET) may offer a promising avenue to visualize and quantify these dynamic processes in vivo, enabling earlier detection, improved risk stratification, and therapeutic monitoring.

**Figure 2:**
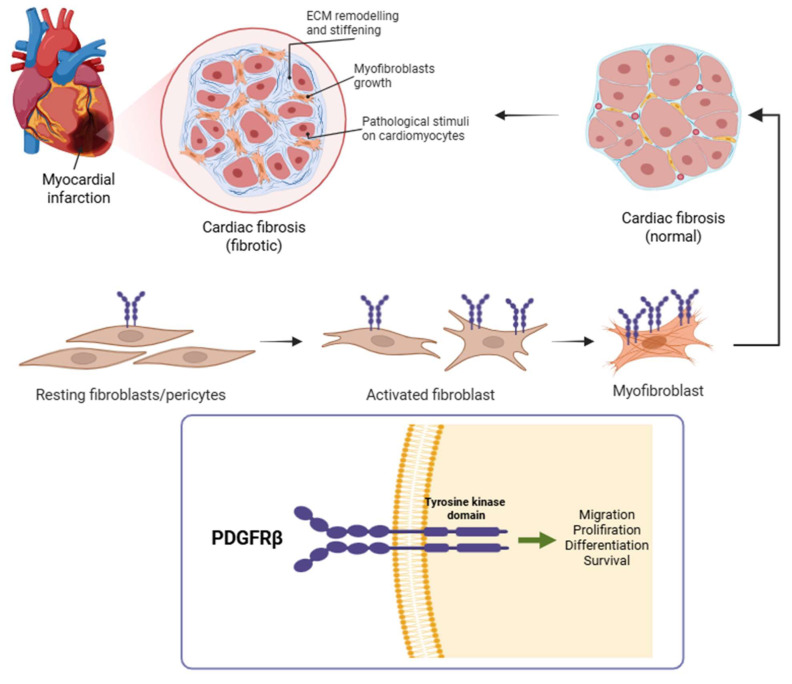
Following myocardial infarction, quiescent cardiac fibroblasts become activated and differentiate into myofibroblasts, which are central to extracellular matrix deposition and scar formation [6]. This panel illustrates that transition, culminating in the fibrotic remodeling of the infarcted myocardium. During this process, PDGFRβ is markedly upregulated on the surface of activated fibroblasts and myofibroblasts. Given its cell-type specificity during active fibrogenesis, PDGFRβ serves as a promising molecular target for non-invasive imaging of myocardial fibrosis. Created with BioRender.com.

Fibrogenesis is driven by the activation and proliferation of cardiac fibroblasts, which differentiate into myofibroblasts and secrete extracellular matrix proteins to form scar tissue (Figure 2) [4]. Monitoring myofibroblast activation may serve as a valuable prognostic and predictive indicator for assessing the effectiveness of treatment following myocardial ischemia–reperfusion (MIR) injury, with the goal of preventing ischemic heart failure. Platelet-derived growth factor receptor beta (PDGFRβ) is a key regulator of this process and is selectively upregulated on activated fibroblasts and pericytes during tissue remodeling. PDGFRβ expression is minimal in quiescent fibroblasts but becomes markedly upregulated upon activation, with even higher levels observed in myofibroblasts. During the regression phase of the disease, the number of myofibroblasts rapidly declines, resulting in reduced PDGFRβ levels in the tissue. Therefore, quantifying PDGFRβ holds promise to monitor the dynamic progression and resolution of fibrogenesis. As such, PDGFRβ represents an attractive molecular target for non-invasive imaging of active fibrosis in the myocardium [5], and other organ systems.

**Figure 3:**
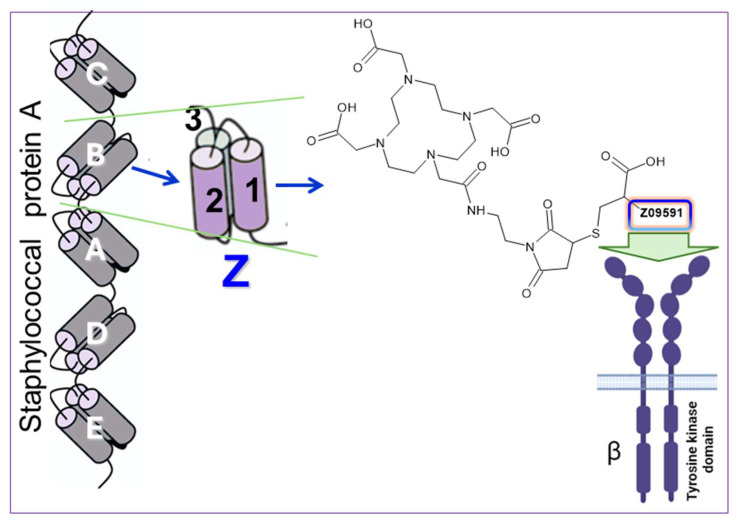
To target PDGFRβ, we used an engineered Affibody molecule derived from the B domain of staphylococcal protein A. Through specific mutations, this domain was transformed into a stable three-helix structure called Z. An analog designated Z09591 was chemically synthesized under GMP conditions and features a C-terminal cysteine to enable site-specific conjugation of a DOTA chelator, forming ATH001. Z09591 binds selectively to PDGFRβ, enabling targeted imaging. Z09591: AENKFNKELIEAAAEIDALPNLNRRQWNAFIKSLVDDPSQSANLLAEAKKLNDAQAPKC.

To image PDGFRβ expression, we developed a novel radiopharmaceutical based on an engineered Affibody molecule, Z09591, with high affinity and selectivity for human PDGFRβ [7]. Affibody molecules are small (∼6.5–7 kDa), protein-based ligands derived from the B-domain of staphylococcal protein A. They are three helical bundle polypeptides engineered for high stability and rapid pharmacokinetics (Figure 3) [8]. These characteristics make them particularly well suited for PET imaging, especially with short-lived and readily available radionuclides such as gallium-68 (t_½_ = 68 min), which allows for high-contrast imaging shortly after injection. By conjugating the Z09591 site specifically with a DOTA chelator and radiolabeling with gallium-68, we generated [^68^Ga]Ga-DOTA-Z09591 ([^68^Ga]Ga-ATH001), a chemically robust, clinically scalable PET tracer for imaging fibrogenic activity via PDGFRβ. In contrast to larger scaffolds such as [^89^Zr]Zr-labeled Fibrobody^®^, which demonstrated high accumulation in fibrotic tissue but suffered from prolonged circulation, elevated off-target uptake in spleen and kidneys, and high physiological liver background [9], the Affibody-based tracer leverages its compact size and rapid clearance to minimize off-target retention and enable same-day imaging.

The Affibody molecule Z09591 has previously been labeled with In-111 [10], Ga-68 [11], and F-18 [12,13] for preclinical evaluation. However, the automated labeling synthesis and production process of a radiopharmaceutical are inherently complex, particularly due to stringent regulatory requirements [14,15]. Therefore, it is imperative to develop a robust and standardized radiolabeling method that can be readily transferred to other medical centers, facilitating the broader clinical implementation of diagnostic methodology.

The aim of the present study was to develop, qualify, validate, and document a GMP- and Good Radiopharmaceutical Practices (GRPP)-compliant production process for [^68^Ga]Ga-ATH001, including preclinical validation in a murine model of myocardial infarction and fibrotic human myocardial tissue as well as a proof-of-concept clinical microdosing PET study in STEMI patients. An advantage of the microdosing approach is that it allows phase 0 clinical studies to be conducted early in the drug development process, thereby reducing clinical trial expenses and supporting timely decisions on the subsequent direction of the development [16,17,18,19,20,21,22]. Although the clinical data in this report are limited to illustrative cases, they demonstrate the tracer’s biological relevance and translational potential. The results establish a foundation for the further clinical development of PDGFRβ-targeted molecular imaging in cardiovascular disease and potentially beyond.

## 2. Results

### 2.1. Production Process Qualification and Validation Outline

The generator was qualified upon delivery, prior to the initiation of production development. Following the completion of the automated labeling synthesis development, but before executing the three consecutive production runs required for process validation, a series of qualification and validation activities were conducted in accordance with current Good Manufacturing Practices (GMP) regulations. In particular, the validation strategy adhered to the principles outlined in the EU Guidelines to Good Manufacturing Practice: Annex I—Manufacture of Sterile Medicinal Products, which underwent substantial revision and entered into force on 25 August 2023 [14,15]. In compliance with these regulatory requirements, bioburden testing was performed during a dedicated production run; sterile filtration validation was completed in one run; and endotoxin testing methods and sterile filter integrity were validated across three production runs each.

Three consecutive process validation batches were subsequently manufactured and assessed according to the acceptance criteria outlined in Table 1, focusing on sterility, residual solvents, endotoxins, and Ge-68 breakthrough. Quality control methods were validated in accordance with specifications presented in Table 2. The process was developed for the production of a ^68^Ga-labeled investigational medicinal product (IMP). All critical quality attributes across the three validation runs met their predefined specifications, as detailed in Table 2 and Table 3. Chemical and radiochemical purity, as well as peptide content, were assessed using UV-Radio-HPLC. The finished product’s stability was evaluated through radiochemical purity testing over a 2 h period at room temperature across all three batches, confirming its integrity throughout the validated production process.

### 2.2. Generator Qualification and Elution Fractionation

The ^68^Ge/^68^Ga generator was qualified to assess key performance parameters, including physical appearance, elution efficiency, and ^68^Ge-breakthrough, along with the tracer labeling capability using validated synthesis protocols aligned with the specifications outlined in the Ph. Eur. monograph [23]. Generator elution was performed automatically using the Modular Lab Pharm platform. The eluate appeared clear and colorless, indicating acceptable visual quality. Radionuclidic purity, assessed by measuring the eluate radioactivity immediately after elution and again 48 h later, once the initial ^68^Ga had decayed to a negligible level, was found to be greater than 99.999%. Elution efficiency was calculated at secular equilibrium, yielding an average of 76.5 ± 2.5%, based on the ratio of recovered ^68^Ga activity to the theoretical activity expected from ^68^Ge decay (with appropriate decay correction applied). To optimize the use of the eluate for radiolabeling, a fractionation protocol was implemented: the first 1.5 mL of eluate was discarded, followed by collection of a 3.5 mL top fraction for synthesis, and a final 2 mL discarded. This 7.0 mL total volume corresponded to the complete generator eluate, with the top 3.5 mL fraction containing 85 ± 2% of the total radioactivity.

### 2.3. Production of [^68^Ga]Ga-ATH001

**Figure 4:**
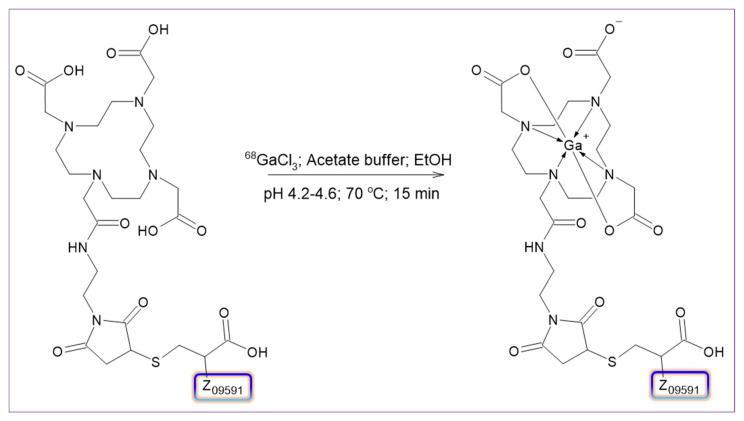
Schematic representation of [^68^Ga]Ga-ATH001 labeling synthesis.

**Figure 5:**
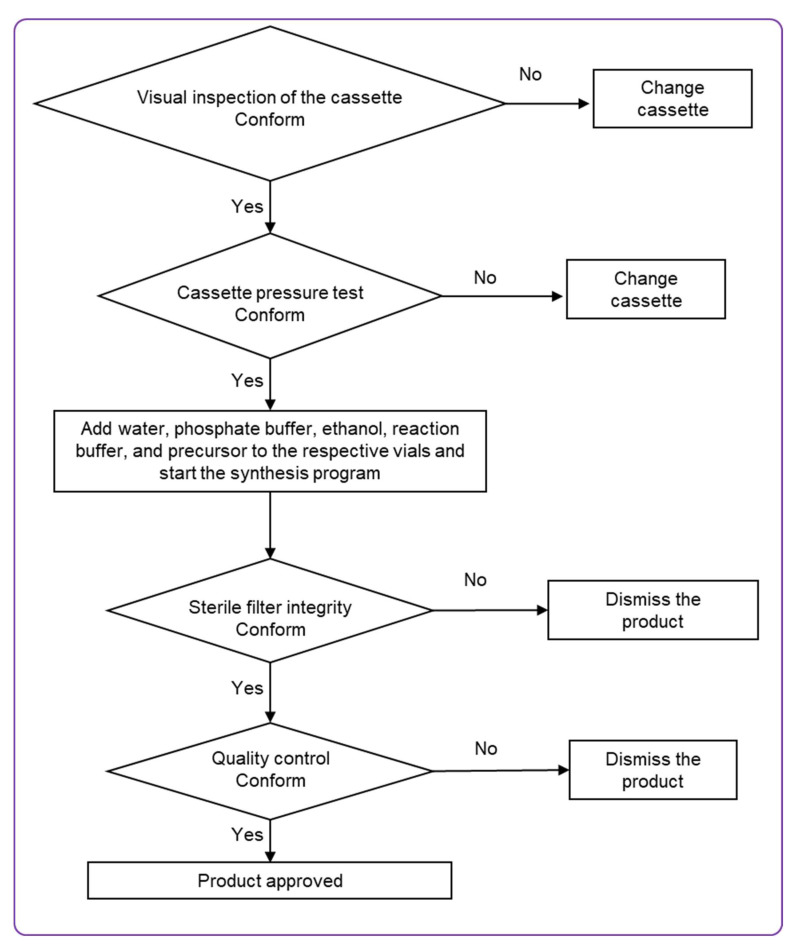
Flow chart of [^68^Ga]Ga-ATH001 production on the Modular-Lab PharmTracer.

The fully automated, cassette-based synthesis of [^68^Ga]Ga-ATH001 (Figure 4) was performed using the commercially available Modular-Lab PharmTracer platform, following the workflow outlined in Figure 5. Single-use, pre-sterilized cassettes eliminated the risk of cross-contamination and ensured compliance with GMP standards.

The production process was carried out in four clearly defined stages. Initially, the integrity of the disposable cassette was verified to ensure it was intact and free of leaks. The second stage involved conditioning the SPE cartridge in preparation for product purification. The third stage encompassed radiolabeling, followed by purification, formulation, and sterile filtration of the final product. The final step focused on verifying the integrity of the sterile filter using a pressure hold test. Overall, the procedure encompassed radiolabeling, purification, and sterile filtration steps. The manufacturing was a continuous process without isolation of the active substance.

The synthesis was based on a fractionation method [24,25,26], and the production was completed within one hour including quality control. Key process parameters such as reaction time, temperature, choice of radical scavenger, buffer concentration, pH, purification strategy, final formulation, and sterile filtration were thoroughly optimized. While Affibody molecules are known to retain structural integrity at temperatures up to 90 °C, the optimal radiochemical yield in this study was consistently achieved at 70 °C. Optimization of the acetate buffer concentration revealed that 0.5 M with pH maintained between 4.2 and 4.6 provided the most robust and reproducible radiolabeling results. Various solid-phase extraction (SPE) cartridges and HPLC columns were assessed to ensure high recovery rates (92.9 ± 2.4%, n = 5; mean ± SD).

**Figure 6:**
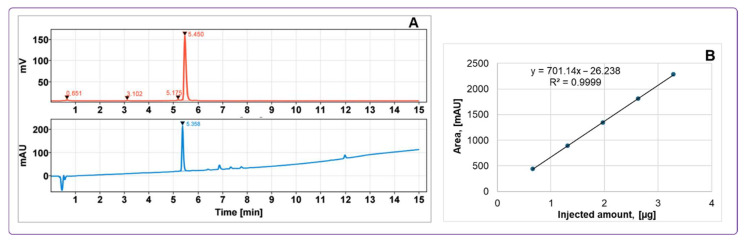
(**A**) Typical HPLC-chromatograms of quality control with UV absorption profile (lower panel) and radioactivity signal profile (upper panel) of [^68^Ga]Ga-ATH001; (**B**) UV-HPLC signal calibration of ATH001 (R^2^ = 0.9999).

The GMP-compliant, reliable, and highly reproducible production process demonstrated a 100% success rate (N = 12) with consistent control over peptide concentration and radioactivity in the final product. The product was eluted from the SPE cartridge using 50% ethanol and subsequently diluted with phosphate buffer (8.8 ± 0.3 mL) during formulation to reduce the final ethanol concentration to below 10%. Final product purification excluded contamination with ^68^Ga and provided radiochemical purity (RCP) of 98.1 ± 0.7% (Figure 6). The molar activity of the tracer at the time of injection ranged from 12 to 24 MBq/nmol dependent on the age of the generator. The total amount of injected peptide was 93 ± 6 µg (n = 4).

**Figure 7:**
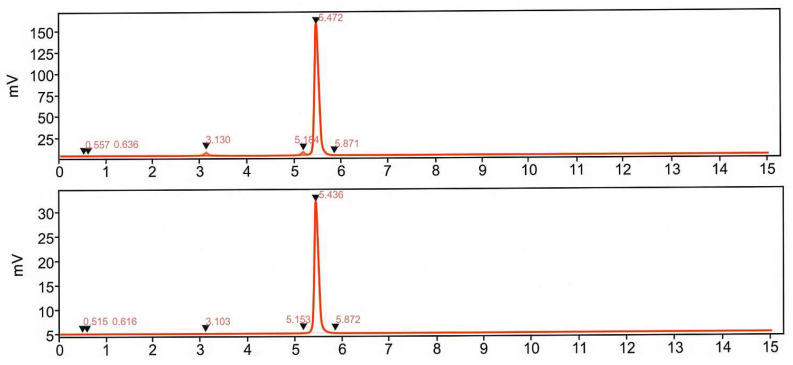
Radio-chromatograms demonstrating influence of ethanol on the formation of the by-products (t_R_ = 3.1 and 5.1 min and intact product (t_R_ = 5.4 min). Upper panel: labeling synthesis without addition of radical scavengers. Lower panel: labeling synthesis with addition of ethanol. The front radiosignal corresponds to non-incorporated ^68^Ga(III).

Decay-corrected radiochemical yield was 61.7 ± 4.9% with a duration of the production of 45–60 min. Stability studies demonstrated that the product remained stable at room temperature for at least 2 h, maintaining a radiochemical purity of 96.8 ± 0.7%. Addition of EtOH (7–10% (*v*/*v*)) as a radical scavenger effectively mitigated oxidative radiolysis and improved RCP (Figure 7). The signals corresponding to the radioactive impurities were reduced by 90%.

A comprehensive summary of the product specifications and validation data is provided in Table 1.

The radiopharmaceutical was formulated in a sterile phosphate buffer containing less than 10% ethanol, with a final volume of 8.8 ± 0.3 mL and a pH of 7. The acceptance criteria required a radiochemical purity greater than 91%, with no individual unidentified radioactive impurity exceeding 5% of the total radioactivity. The product was sterilized by filtration through a 0.22 µm membrane filter. The final product was supplied in a sterile, capped 10 mL glass vial, labeled with a unique batch number and relevant product information. Accurate quantification of the administered radioactivity and peptide dose was achieved by measuring residual radioactivity in all syringes and containers before and after administration, combined with UV-HPLC analysis to determine peptide concentration (Figure 6).

### 2.4. Quality Control

The HPLC method for evaluating the radiochemical purity and concentration of [^68^Ga]Ga-ATH001, using both UV and radio detection, was comprehensively validated. Key parameters included specificity, linearity, repeatability, radioactivity recovery from the HPLC column, and product stability at room temperature (Table 2). UV signal calibration enabled accurate quantification of peptide content, yielding a Pearson correlation coefficient of R^2^ = 0.9999 (Figure 6), with a concentration range consistent with expected product levels.

To confirm specificity, the method ensured effective retention and separation of the tracer from impurities, buffers, and excipients in the formulation. The developed HPLC protocol provided baseline separation between oxidized and non-oxidized forms, ensuring distinct retention times. Detection limits were evaluated to confirm sufficient UV signal intensity for both peptide quantification and corresponding radio signal identification (Figure 6). Repeatability was demonstrated through six injections of a standard solution at product-relevant concentrations, with results meeting acceptance criteria. Column recovery studies showed over 99% of radioactivity was eluted from the HPLC column, confirming accurate radiochemical purity assessment. Overall, specificity, linearity, and precision were validated for both UV and radio detectors, supporting the method’s robustness for routine quality control. The tests and methods are summarized in Table 3.

### 2.5. In Vitro Autoradiography Binding Assay of [^68^Ga]Ga-ATH001 in Mice with Ischemic Heart Injury

#### 2.5.1. Murine Infarcted Myocardium

**Figure 8:**
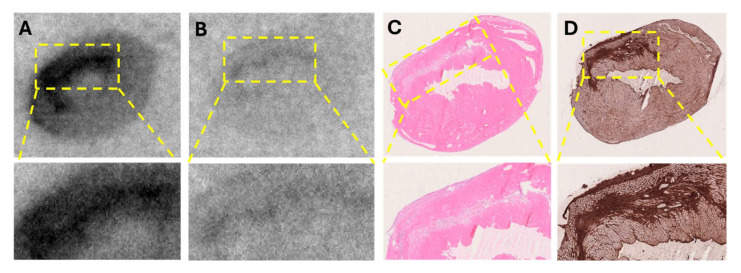
In vitro detection of fibrotic activity using [^68^Ga]Ga-ATH001 in a murine model of myocardial ischemia–reperfusion (MIR) injury. Representative autoradiograms of total binding of [^68^Ga]Ga-ATH001 (5 nM) to PDGFRβ (**A**) and blocking of the binding to PDGFRβ with ATH001 (1.2 µM) (**B**); Representative images of H&E staining showing cell damage (**C**), and immunohistochemistry of PDGFRβ (**D**).

The in vitro autoradiography assay demonstrated a clear increase in [^68^Ga]Ga-ATH001 binding in the infarcted areas (Figure 8A). The localization of the infarct was confirmed by H&E and SR (Figure 8C,D), as well as the upregulation of PDGFRβ protein by IHC (Figure 8D. Competition with unlabeled ATH001 in excess almost completely abolished the binding, indicating receptor-specific binding (Figure 8B). Interestingly, a smaller, but detectable blockable signal was also observed in the remote and contralateral regions of the left ventricle, perhaps indicative of upregulation of PDGFRβ in response to the infarction.

#### 2.5.2. Human Fibrotic Myocardial Tissue

**Figure 9:**
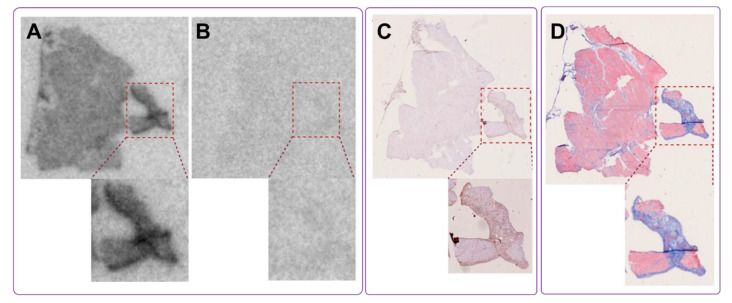
In vitro autoradiography of fibrotic tissue from a human heart, demonstrating specific uptake of the radiopharmaceutical in fibrotic regions (**A**). Binding was significantly reduced in the presence of excess unlabeled precursor, confirming target specificity (**B**). The tracer binding co-localized with fibrotic regions identified by anti-PDGFRβ IHC (**C**) and Masson’s trichrome staining (**D**), which highlights extensive collagen deposition in the same areas, indicating mature fibrosis.

Histopathological examination by pathologist confirmed moderate myocardial scarring with fibrotic loci in the biopsy. The autoradiography assay revealed heterogeneous but distinct uptake of [^68^Ga]Ga-ATH001 (Figure 9A). Pre-incubation of adjacent sections with unlabeled ATH001 led to an almost total abolishment in the tracer signal, indicating high specificity (Figure 9B). The high-intensity binding areas corresponded well with the localization of collagen-rich fibrotic foci as assessed by MTC (Figure 9D). Similarly, immunostaining confirmed the upregulation of PDGFRβ in areas with high specific binding of [^68^Ga]Ga-ATH001.

### 2.6. Clinical Imaging in STEMI Patients

**Figure 10:**
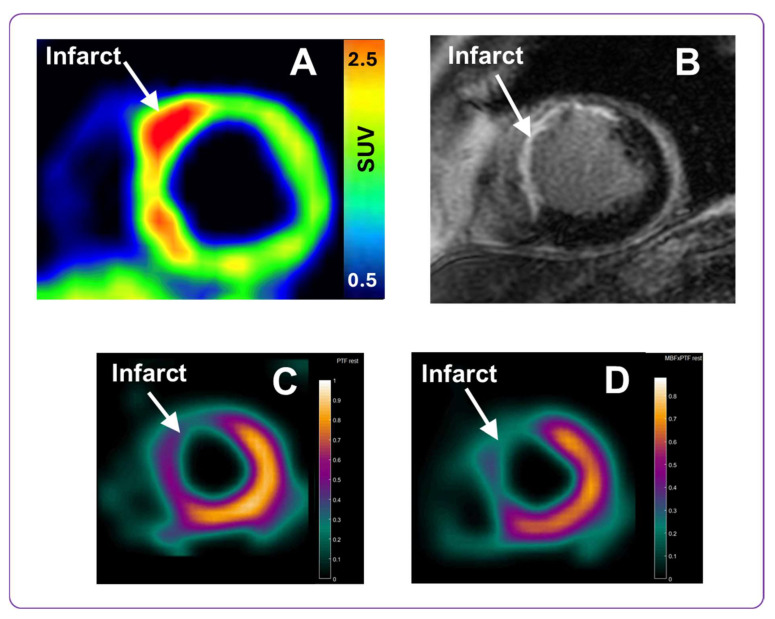
Representative PET and MRI images obtained using the PDGFRβ-targeted tracer [^68^Ga]Ga-ATH001, and gadolinium contrast agent, respectively. The upper panel presents the [^68^Ga]Ga-ATH001 PET scan normalized to 0.5–2.5 SUV (**A**) and gadolinium late enhancement MRI (**B**) images from a patient one week after PCI for STEMI. The lower panel presents parametric ^15^O-H_2_O images of Perfusable Tissue Fraction (PTF) (**C**) and the Myocardial Blood Flow (MBF) (**D**) from the same patient.

Here, we present preliminary images of [^68^Ga]Ga-ATH001 uptake in a patient with STEMI, and its correlation to the location of the infarct and the associated perfusion defect. The images will later be quantified when the entire study with eight patients in each cohort have been examined and will be presented separately. The scans presented qualitatively herein were performed 1 week post PCI. The injected mass of the tracer was below 100 µg per subject, and the injected radioactivity was 160 MBq. No adverse events related to the tracer were observed at these dosing levels. The patient demonstrated strong focal [^68^Ga]Ga-ATH001 uptake localized to the infarct regions (Figure 10A), as detectable by gadolinium late enhancement MRI, indicating extracellular volume expansion (Figure 10B). However, the tracer uptake was also increased in the peri-infarcted regions beyond the verified infarct. Uptake in the right ventricle was low (Figure 10A, seen as a faint blue signal to the left of the panel). The area with high [^68^Ga]Ga-ATH001 binding also overlapped well with a perfusion defect in the myocardium seen in by ^15^O-H_2_O (Figure 10C,D).

## 3. Discussion

The development of [^68^Ga]Ga-ATH001, a PDGFRβ-targeted Affibody molecule-based PET tracer, addresses an unmet clinical need for non-invasive imaging of active fibrogenesis in patients following myocardial infarction (MI). Unlike current clinical tools that primarily assess structural or functional consequences of fibrosis, this radiopharmaceutical enables visualization of PDGFRβ expression, a hallmark of myofibroblast activation, offering a mechanistically targeted approach to detect fibrotic activity at earlier stages of disease. PDGFRβ is upregulated on activated fibroblasts and pericytes during tissue remodeling and fibrogenesis, not only in the infarcted heart but also in other organs such as the liver, lungs, and kidneys [4,27].

The Affibody molecule Z09591 was specifically selected and engineered for high-affinity binding to human PDGFRβ, incorporating a C-terminal cysteine to enable site-specific conjugation to DOTA chelator. The resulting compound, ATH001, exhibits favorable pharmacokinetic properties including high in vivo stability, low immunogenicity, hydrophilicity, and rapid renal clearance with minimal non-specific uptake. These features, together with its robust and automatable radiolabeling protocol using commercially available synthesis cassettes, support reliable production of [^68^Ga]Ga-ATH001 and facilitate multi-center clinical translation.

[^68^Ga]Ga-ATH001 holds promise not only for early identification of patients at risk of adverse cardiac remodeling, arrhythmogenic substrate formation, or poor response to reperfusion therapy, but also as a tool for monitoring anti-fibrotic or anti-inflammatory interventions. Quantitative PET readouts of PDGFRβ expression may ultimately serve as both a diagnostic biomarker and a pharmacodynamic endpoint in clinical trials targeting myocardial fibrosis. Building on the broader potential of molecular imaging to move beyond anatomical assessments, this work highlights the capacity of [^68^Ga]Ga-ATH001 to capture dynamic, biologically relevant processes central to cardiac disease progression.

### 3.1. Radiochemistry and Production

The qualification of the ^68^Ge/^68^Ga generator is a fundamental step in ensuring the consistent production of a high-quality radiopharmaceutical. As the generator is directly involved in the manufacturing process, it must comply with rigorous standards to assure product quality, patient safety, traceability, and robust performance. The qualification and validation of the chromatographic generator include detailed investigations of its elution profile and efficiency, radionuclidic purity to limit ^68^Ge breakthrough, as well as potential contamination from metal cations and column matrix material. These assessments follow the European Pharmacopoeia monographs on Gallium (^68^Ga) chloride solution and Gallium (^68^Ga) edotreotide injection [23,28], which establish the key regulatory framework governing generator use in clinical radiopharmaceutical production. In this study, the GalliaPharm^®^ generator, classified as a medicinal product, met the Ph. Eur. monograph specifications, and therefore did not require additional validation. Nonetheless, ^68^Ge breakthrough monitoring was conducted during the three process validation productions (and periodically thereafter), and the elution profile was optimized to select the top fraction with the highest ^68^Ga concentration. The synthesis program was accordingly adjusted to maximize radiochemical yield. To reduce metal cation impurities and ensure reproducibility, the generator was routinely eluted 2–24 h prior to synthesis, a well-established strategy to enhance labeling efficiency and minimize interference with chelation chemistry [24].

Automation of the production process was essential to minimize radiation exposure to personnel, enhance process robustness, and reduce the risk of human error, which are critical factors in clinical environments involving patient studies [29]. In addition to improving safety and reliability, automated synthesis provides real-time documentation for Good Manufacturing Practice (GMP) compliance, which is vital for clinical-grade radiopharmaceutical production. The use of a commercially available automated platform facilitates straightforward technology transfer to other sites, supporting standardization and harmonization across multicenter clinical trials [30,31,32,33,34]. This accelerates regulatory approvals and paves the way for routine clinical implementation of [^68^Ga]Ga-ATH001 PET-CT diagnostics. Importantly, this production method builds on the established synthesis protocol for [^68^Ga]Ga-ABY025 [26] which led to the development of standardized disposable cassettes and integrated software now available commercially. This continuity supports broader dissemination of [^68^Ga]Ga-ATH001 for theranostic applications worldwide.

The fully automated, cassette-based synthesis of [^68^Ga]Ga-ATH001 was performed using the Modular-Lab PharmTracer platform. This system employs single-use cassettes to eliminate cross-contamination, enable high-throughput synthesis, and ensure reproducibility. It supports common radiolabeling techniques, including generator eluate fractionation and pre-concentration. In this study, the fractionation method was used [24,25,26] as it aligns with the previously validated [^68^Ga]Ga-ABY025 protocol. To adapt the method to different medical sites, only minimal adjustments such as primarily optimization of the eluate fraction and flow rate that are required, depending on the generator model and tubing configuration. For instance, the top fraction volume collected for the labeling synthesis was increased from 1–1.5 mL [26] to 3.5 mL in the production of [^68^Ga]Ga-ATH001. This adjustment was necessary because the full available radioactivity was used in the synthesis, unlike in the case of [^68^Ga]Ga-ABY025, where a lower amount of radioactivity was employed to ensure that the entire final product could be administered in a single injection. Thus, given that the necessary hardware, cassettes, and software are commercially available, technology transfer and local implementation can be achieved with minimal effort.

Given the favorable imaging characteristics of [^68^Ga]Ga-ATH001 even at lower specific activity, the simpler fractionation method was selected for automation. Although this approach requires a larger amount of precursor, it eliminates the need for additional buffers and reduces both the risk of human error and overall processing time. Notably, radioactivity losses associated with pre-concentration methods, due to irreversible retention on ion-exchange cartridges and extended processing times, can be comparable to, or even exceed, the approximately 17% loss observed with the fractionation method. One of the primary advantages of the pre-concentration method [24,25] is its ability to reduce the generator eluate volume, thereby allowing the use of minimal precursor amounts to achieve high specific activity. However, since a sufficiently high amount of precursor was used in this study and high specific activity was not a critical requirement, the simpler fractionation method was deemed more appropriate. Various solid-phase extraction (SPE) cartridges for the product purification and HPLC columns for the product quality control were evaluated to ensure optimal recovery of the product. SPE cartridges achieved an average recovery of 92.9 ± 2.4%, supporting high product yield and consistent reproducibility. The HPLC column demonstrated complete recovery, enabling reliable quality control analysis.

To prevent direct exposure of the protein to the acidic generator eluate, which can cause oxidation or degradation, the eluate was first mixed with reaction buffer prior to transfer into the precursor-containing reactor [26,35]. Since the protein contains tryptophan residues that are particularly susceptible to radiolytic oxidation, ethanol was added to the reaction mixture. This effectively suppressed oxidation by-product formation to below 1%, and eliminated the need for additional radical scavengers such as ascorbic acid or dihydroxybenzoic acid [25]. Ethanol offered multiple advantages in this process: it is biocompatible, GMP-compatible, non-toxic, and non-immunogenic. It does not interfere with the radiolabeling reaction or target binding and can also improve the solubility of lipophilic precursors. Importantly, ethanol addition enhanced the radiochemical purity of the final product. During purification and formulation, ethanol concentration was diluted to below 10%. The final radiopharmaceutical formulation remained chemically stable at room temperature for at least 2 h, with no detectable degradation.

Quality control methods for determining radiochemical purity and protein content were fully validated using UV-radio HPLC, demonstrating high specificity, linearity, and repeatability. These validated protocols ensured robust and reliable quality and accurate quantification throughout the production process, which is critical for accurate patient dosing and consistent interpretation of imaging data, especially in the context of clinical trials where precise quantification is essential. Product identity was confirmed by comparing the retention time of [^68^Ga]Ga-ATH001 to that of the metal-free precursor ATH001 using UV-radio HPLC. The known delay between UV and radioactivity detectors was taken into account during this comparison. The metal-free precursor was also employed before final product analysis to verify HPLC system performance, perform single-point calibration, and prevent signal overlap that could interfere with UV-based quantification of the radiolabeled product. Accurate determination of the protein amount administered to the subjects was achieved through validated UV-HPLC analysis, UV-calibration curves, and assessment of radioactivity distribution across vials and syringes during the administration. This approach ensured precise protein quantification and dosing for clinical PET imaging applications [26].

### 3.2. Regulatory Aspects and GMP-Compliance

Due to the short half-life of ^68^Ga, it is not feasible to complete full release testing, such as ^68^Ge breakthrough, residual solvent content, and sterility, prior to patient administration. Therefore, in accordance with the extemporaneous preparation approach permitted under GMP for short-lived radiopharmaceuticals, these parameters were controlled during process validation and monitored periodically thereafter. This underscores the necessity for a fully qualified and reproducible production method with validated quality control procedures and comprehensive batch documentation to ensure patient safety and regulatory compliance.

The primary objectives of Good Manufacturing Practice (GMP) are to ensure product quality, subject safety, traceability, and the reliability of the manufacturing process. For radiopharmaceuticals, these standards must be met while considering the unique characteristics of these agents, including their short half-life and sub-pharmacological dosing. Since radiopharmaceuticals are typically administered in microdoses, the associated risk to human subjects is substantially lower than that of conventional therapeutic agents, particularly in early-phase clinical trials. This has been acknowledged by regulatory authorities such as the EMA and FDA, both of which recognize the microdosing concept, defined as ≤100 µg or ≤30 nanomoles for peptides and proteins, and have introduced corresponding guidelines, such as the Exploratory Investigational New Drug (eIND) framework [16,17,18,36]. These regulatory provisions reduce the scope of preclinical toxicity testing required for PET tracers, thereby lowering the overall cost and complexity of development while still ensuring safety and efficacy [30,31]. The inherently high sensitivity of PET imaging supports the use of such low, non-pharmacologically active doses. Moreover, adverse reactions to PET radiopharmaceuticals are exceedingly rare, with no serious or life-threatening events reported [37]. Additionally, Annex 3 of Eudralex provides some flexibility by recognizing that alternative manufacturing approaches may be acceptable, if they align with core GMP principles, further supporting innovation and adaptation in radiopharmaceutical production.

Despite these regulatory adaptations, the development and clinical implementation of radiopharmaceuticals remain constrained by a dual regulatory framework that requires adherence to both pharmaceutical manufacturing standards and radiation safety regulations. This complexity substantially slows innovation and limits broader clinical adoption. A significant challenge is compliance with GMP, particularly following the enforcement of the revised EU GMP Annex I as of 25 August 2023 [14,15]. The updated Annex I introduces more stringent requirements for aseptic processing, environmental monitoring, and contamination control, challenges that are especially difficult for radiopharmaceuticals due to the need for rapid synthesis and immediate use, minimal operator intervention to limit radiation exposure, and the often-limited infrastructure and specialized staff in academic or smaller clinical settings. These enhanced standards significantly increase the operational and financial demands, making compliance difficult for many small-scale producers and complicating early-phase clinical trials. Consequently, Annex I has raised the threshold for clinical-grade production, further delaying the translation of novel tracers into clinical practice.

This context underscores the importance of our work in developing a robust, GMP-compliant radiopharmaceutical production method that is readily transferable to other laboratories. The use of commercially available equipment further supports standardization, regulatory alignment, and broader clinical adoption.

In accordance with the updated regulatory requirements outlined in Annex I of the EU GMP guidelines, the validation of the production process was expanded to include a comprehensive sterility assurance framework. The revised Annex I introduces a risk-based, lifecycle-oriented approach to contamination control and sterility assurance. These principles are particularly challenging to implement in radiopharmaceutical production, where short half-lives, small batch sizes, and accelerated manufacturing timelines limit the flexibility typically available in conventional pharmaceutical processes.

Before initiating the formal validation runs, key qualification and validation activities were conducted to ensure process robustness and regulatory compliance. The gallium generator was qualified upon delivery, prior to any production development. The automated labeling synthesis underwent method qualification, establishing that it could consistently produce a product meeting predefined quality specification. Sterility-related validation steps included bioburden testing performed during a dedicated production run to verify microbial control before sterile filtration. Sterile filtration validation was completed in a single run and included mandatory pre- and post-use integrity testing of the filter to confirm performance and detect potential breaches. In addition, endotoxin testing and sterile filter integrity methods were validated across three production runs, in alignment with Annex I expectations for quantifiable detection sensitivity and lifecycle-based assurance of contamination control.

Following these preliminary validation steps, three consecutive full-scale process validation batches were manufactured and evaluated according to predefined acceptance criteria (Table 1), focusing on sterility, residual solvents, endotoxins, and Ge-68 breakthrough. The validated process was developed specifically to produce a [^68^Ga]-labeled investigational medicinal product (IMP). Across all three validation batches, all critical quality attributes (CQAs) met their acceptance criteria (Table 1). The stability of the final product was confirmed through radiochemical purity testing over a 2 h period at room temperature, demonstrating consistent product integrity throughout the validated process.

### 3.3. Preclinical Evaluation

The GMP-produced [^68^Ga]Ga-ATH001 was subjected to preclinical evaluation to confirm that its biological properties were retained, as the production process differed substantially from the earlier manually performed synthesis. The earlier approach involved smaller volumes, lower radioactivity levels, minimal handling, and different materials, which reduced the risk of radiolysis and simplified processing. In contrast, the automated GMP-compliant process introduced higher radioactivity concentrations, increased transfer steps, and more complex workflows. To ensure that these changes did not affect the tracer’s integrity and biological activity, we conducted preclinical assays on the GMP-grade product described herein. The results confirmed that both specificity and binding affinity were preserved.

In a murine model of myocardial infarction, the tracer demonstrated target-specific binding, with in vitro autoradiography showing strong uptake in the infarcted region. This signal was significantly reduced by pre-incubation with excess unlabeled compound, confirming specificity for PDGFRβ. Co-localization with SR staining, H&E staining, and PDGFRβ immunostaining further validated the tracer’s accumulation in fibrotic areas undergoing active tissue remodeling. These results not only confirmed the biological performance of the GMP-produced tracer but also provided strong support for its clinical translation.

The in vitro autoradiography using human myocardial tissue also provided compelling evidence for the specific binding of [^68^Ga]Ga-ATH001 to fibrotic regions. Using cryosections from infarcted human hearts, we demonstrated that tracer uptake was markedly reduced when adjacent sections were pre-saturated with excess unlabeled precursor, confirming receptor-mediated binding. This specific displacement in relevant human tissues strongly supports the tracer’s selective interaction with its intended molecular target. Importantly, the spatial distribution of [^68^Ga]Ga-ATH001 signal correlated closely with histopathological markers of myocardial fibrosis. MTC staining revealed substantial collagen deposition in the infarcted myocardium, and immunohistochemical analysis confirmed the presence of PDGFRβ within these fibrotic regions. The close co-localization between radiotracer binding and PDGFRβ expression, even at higher magnification, further underscores the biological relevance and molecular specificity of the tracer.

These findings validate the targeting strategy of [^68^Ga]Ga-ATH001 and provide a critical translational link between preclinical studies and its intended clinical application. Early clinical data further demonstrated excellent imaging characteristics, reinforcing the potential of [^68^Ga]Ga-ATH001 for non-invasive assessment of fibrotic activity.

### 3.4. Initial Clinical Results

The initial clinical use of [^68^Ga]Ga-ATH001 demonstrated excellent performance, supporting its continued development and broader application in cardiac imaging. To assess the tracer’s translational potential, representative cases from an ongoing phase 0 microdosing study were included. Although not the primary focus of this manuscript, these examples offer compelling evidence of the tracer’s in vivo specificity and feasibility. A pilot clinical study was conducted in patients with STEMI who were imaged one week after PCI. No adverse events related to the tracer were reported, confirming its favorable safety profile.

STEMI patients demonstrated focal tracer uptake localized to infarct zones, which were also identified by gadolinium-enhanced MRI and ^15^O-H_2_O PET perfusion imaging. The observed uptake was the highest in infarcted areas but was also present in the adjacent peri-infarct regions. These initial results are promising. Compared to other fibrotic imaging agents, such as fibroblast activation protein (FAP) ligands, [^68^Ga]Ga-ATH001 targets PDGFRβ, which is more selectively expressed on activated fibroblasts and pericytes, reducing non-specific uptake from inflammatory or epithelial cells. Additionally, the Affibody scaffold offers fast pharmacokinetics and a short imaging window compatible with ^68^Ga half-life (68 min), allowing same-day imaging and streamlined clinical workflows.

## 4. Materials and Methods

### 4.1. Materials, Equipment, and Facilities

The DOTA-Cys-ATH001 peptide precursor, serving as the active pharmaceutical ingredient (API) starting material, was chemically synthesized by Almac in accordance with current Good Manufacturing Practice (cGMP) standards and supplied by Antaros Tracer AB. The Affibody molecule, which specifically targets PDGFRβ, was selected for its previously established high affinity and selectivity toward the human PDGFRβ receptor. A cysteine residue was introduced at the C-terminus to enable site-specific conjugation with the DOTA chelator. The resulting construct, termed ATH001, was produced via solid-phase peptide synthesis (SPPS) under GMP-compliant conditions and subsequently purified using high-performance liquid chromatography (HPLC). Product identity and purity were verified through analytical HPLC and mass spectrometry. Manufacturing was carried out in accordance with international cGMP standards, including EU directive 2003/94/EC, ICH Q7 guidelines for APIs, and U.S. regulations 21 CFR Parts 210 and 211. Preclinical studies in animal models found no toxicity attributable to DOTA-Cys-ATH001. The manufacturer provided both certificates of compliance and certificates of analysis. The product, DOTA-Cys-ATH001, with a molecular weight of 7.06 kDa, was supplied in lyophilized form in Ph.Eur. type I glass vials sealed with rubber stoppers and aluminum caps. Each vial contained 400 µg of sterile, non-pyrogenic, preservative-free DOTA-Cys-ATH001.

All reagents used in the preparation process were of analytical or pharmaceutical grade and used without additional purification. These included ultrapure HCl (Merck, Darmstadt, Germany), sodium acetate buffer at pH 4.6 (Sigma-Aldrich, Stockholm, Sweden), sterile water (Fresenius Kabi, Uppsala, Sweden), phosphate buffer (Apoteket AB, Solna, Sweden), 10 M NaOH (Sigma-Aldrich, Stockholm, Sweden), ethanol (APL), high-purity water (TraceSelect, Fluka, Buchs, Switzerland), and trifluoroacetic acid (Merck, Darmstadt, Germany).

Aseptic labeling synthesis of [^68^Ga]Ga-ATH001 was carried out within a GMP-compliant Grade A laminar airflow workstation (LAFW), located in a cleanroom environment classified as Grade C according to GMP standards. Both the ^68^Ge/^68^Ga generator (GalliaPharm^®^, Eckert & Ziegler Radiopharma GmbH, Berlin, Germany) and the Modular-Lab PharmTracer automated synthesis unit (Eckert & Ziegler Eurotope GmbH, Berlin, Germany) were installed inside the LAFW to maintain sterility throughout the radiolabeling process. Product quality control was performed using a high-performance liquid chromatography (HPLC) system (LaChrom, Hitachi, Tokyo, Japan), which included a Hitachi Chromaster 5110 pump (model L-2130, Tokyo, Japan), a Knauer UV detector (model 40D; L-2400, Tokyo, Japan), and a radiation flow detector (Bioscan, Washington, DC, USA) arranged in series. Analyte separation was achieved on a reversed-phase analytical column (Accucore 150-C4, 50 × 3.0 mm, 2.6 µm particle size). The mobile phase consisted of solvent A (10 mM trifluoroacetic acid in water) and solvent B (acetonitrile with 10 mM trifluoroacetic acid). UV absorbance was monitored at 220 nm. The elution followed a linear gradient: from 10% to 80% B over 0–12 min, then from 80% to 90% B over 12–15 min. The flow rate was maintained at 1.0 mL/min. Data acquisition and processing were carried out using the EZChrom Elite software (v. 3.3, Santa Clara, CA, USA). 

The production utilized gallium-68 (^68^Ga; half-life = 68 min; β^+^ emission = 89%; electron capture = 11%) as the radionuclide source, which was obtained by eluting a pharmaceutical-grade ^68^Ge/^68^Ga generator (1850 MBq, GalliaPharm^®^, Berlin, Germany) with 0.1 M hydrochloric acid. According to the manufacturer’s specifications, the levels of metallic impurities were below the maximum thresholds defined by the European Pharmacopeia monograph [28]. The ^68^Ga eluate appeared clear and colorless upon visual inspection. The level of ^68^Ge breakthrough in the eluate was determined as a percentage of the total ^68^Ga radioactivity by analyzing aliquots from the generator eluate. Initial measurements were performed immediately after elution using an ionization chamber coupled with a NaI(Tl) scintillation detector, and residual radioactivity was assessed 48 h later with a well-type NaI(Tl) scintillation counter.

### 4.2. Production and Quality Control of [^68^Ga]Ga-ATH001

The radiolabeling process was carried out using a commercially available, fully automated synthesis platform (Modular-Lab PharmTracer, Berlin, Germany) equipped with a sterile, single-use cassette system (AS-GA-ABY025-C4-1.0). The automated sequence comprised a cassette pressure check, radiolabeling reaction, purification, formulation, sterile filtration, and a subsequent integrity test of the sterilizing filter. Critical parameters such as reaction time, temperature, and radioactivity levels were continuously monitored in real time. The protocol also incorporated a generator eluate fractionation step. For purification, an Oasis HLB solid-phase extraction (SPE) cartridge was employed, and the final radiolabeled product was eluted using 1 mL of 50% ethanol.

The cassette was loaded with a reaction buffer consisting of 700 µL of 0.5 M sodium acetate buffer containing 4% of 10 M sodium hydroxide and 27% ethanol, ATH001 precursor solution (780 µL, 30 nmol), sterile water, phosphate buffer, and 50% ethanol solution. The radiolabeling reaction was carried out by heating the mixture at 70 °C for 15 min. Upon completion of the reaction, the crude product was cooled by the addition of 3.5 mL of water and subsequently purified using a solid-phase extraction (SPE) cartridge (Oasis HLB). The final product was eluted from the cartridge using 1 mL of 50% ethanol. Following purification, the product was diluted with phosphate-buffered solution to a total volume of 8.8 ± 0.3 mL for the formulation. It was then sterile filtered through a 0.22 µm filter into a sterile, 10 mL glass vial sealed with a sterile cap. The integrity of the sterilizing filter was verified directly on the synthesis platform using pressure hold test. A sample of the final formulation was taken for analysis to determine identity, radiochemical purity, peptide concentration, and pH. The total radioactivity of the finished product was measured using a dose calibrator.

The chemical purity, radiochemical purity, and peptide content were assessed using high-performance liquid chromatography (HPLC). During the process validation, a portion of the final product was reserved for subsequent analysis of ^68^Ge breakthrough, residual solvents, sterility, and endotoxin levels. Product stability at room temperature was monitored over a 2 h period using UV-Radio-HPLC. The HPLC method was validated to confirm specificity, linearity, and precision in terms of repeatability. The recovery of radioactivity from the analytical HPLC column was evaluated to ensure that no radiolabeled product or radioactive impurities remained on the column. This was performed by collecting the entire HPLC effluent from three replicate runs. For comparison, the column was replaced with a piece of tubing, and the sample was reinjected, collecting the same effluent volume as with the column in place. The radioactivity of all collected fractions (with and without the column) was measured using a well-type NaI(Tl) scintillation counter, with corrections applied for dead-time, decay, and volume. These experiments were conducted for both the radiolabeled product ([^68^Ga]Ga-ATH001) and free ^68^Ga(III).

### 4.3. In Vitro Autoradiography Binding Assay

An autoradiography assay was developed to investigate the binding of [^68^Ga]Ga-ATH001 on myocardial tissue sections of murine and human origin. The murine sections were obtained from animals with myocardial infarction (ethical permit 5.8.18-1434.2023). Briefly, an ischemia–reperfusion myocardial injury was induced in female NMRI mice by temporarily ligating the left anterior descending (LAD) coronary artery for 40 min according to standard practice. After 14 days, the mice were euthanized and the heart snap-frozen and embedded in OCT. For the current study, the entire mouse heart was cryo-sectioned (20 µm thickness) from the apex to the base and placed on Superfrost+ object glasses. Sections comprising an infarcted area were selected for binding studies. Frozen human cardiac tissue samples from patients with myocardial fibrosis (n = 3, confirmed by pathologist) were obtained from the Uppsala Biobank under ethical approval 2024-00969, granted by the Swedish Ethical Review Authority. All specimens were collected from the apical region of the left ventricle at the time of left ventricular assist device implantation, with prior informed consent for future research use. Tissues were cryo-sectioned at 20 µm thickness for autoradiography and 4 µm for histological analysis and placed on Superfrost+ object-glass.

Tissue sections were incubated with the tracer post-excision, a procedure commonly referred to as in vitro autoradiography in radiopharmaceutical development. In line with conventional biological terminology, this can also be described as ex vivo incubation, since the tissue was isolated from a living organism. Autoradiographic binding studies were performed by incubating serial 20 µm sections of either murine or human origin with 5 nM [^68^Ga]Ga-ATH001 (approximately 0.05 MBq/mL) in phosphate-buffered saline (PBS) containing 1% bovine serum albumin (BSA) at room temperature for 60 min. BSA was added to reduce non-specific background binding. To assess binding specificity, blocking studies were performed by co-incubating serial sections with a 100-fold molar excess of unlabeled ATH001. After incubation, tissue sections were washed sequentially in PBS with 1% BSA (1 × 1 min), PBS alone (2 × 1 min), and Milli-Q water, then air-dried at 37 °C. Samples were exposed to phosphor imaging plates (Cytiva) for >6 h alongside standards of known activity for quantification and digitalized using a Typhoon FLA 7000 phosphorimager (Cytiva). Autoradiograms were visualized using the ImageJ software (v. 1.53, NIH).

### 4.4. Histological Assessment of Fibrosis and PDGFRβ Expression

All histological and immuno-staining was performed by the pathology core laboratory at Uppsala University Hospital according to standard methods and the manufacturer’s protocol. For murine tissues, cryosections from the similar region as evaluated by autoradiography were stained for hematoxylin/eosin (H/E), Sirius Red (SR) or PDGFRβ (dilution 1:300, ab32570, Abcam, Cambridge, UK). For the human section, serial sections were stained for Masson’s Trichrome (MTC) or PDGFRβ (dilution 1:300, ab32570, Abcam). The staining protocol was described in detail previously [13]. The processed sections were digitalized by a NanoZoomer S60 slide scanner (Hamamatsu) at 20× magnification and visualized using the QuPath software (Version 0.2.3).

### 4.5. Clinical PET/MRI Study

The initiated observational phase 0 clinical study (ClinicalTrials.gov ID: NCT06956560) received regulatory approval from Swedish Ethical Review Authority. All participants provided written informed consent. Patients with ST-elevation myocardial infarction (STEMI) were recruited at Uppsala University Hospital. Eligibility criteria included identification of a culprit lesion in a proximal coronary artery following percutaneous coronary intervention (PCI), with inclusion limited to individuals over 40 years of age, regardless of gender. Exclusion criteria comprised standard contraindications for MRI and PET imaging, such as claustrophobia, metallic implants, or pregnancy.

Briefly, enrolled subjects were stratified into two cohorts based on post-PCI levels of NT-proBNP: patients with levels exceeding 500 pg/mL were categorized as high risk (cohort 1, current status n = 3, planned for n = 8), while those with levels below 500 pg/mL were assigned to the low-risk group (cohort 2, current n = 3, planned for n = 8). Each patient underwent two imaging sessions using [^68^Ga]Ga-ATH001 PET/MRI: the first at one week post-PCI and the second at two months. Here, results from the first session are presented as preliminary proof-of-concept. A detailed quantification of the entire data set will be presented when the study is completed.

The PET/MRI scanning protocol was designed as follows: first, a ^15^O-H_2_O myocardial perfusion scan was performed (target dose 400 MBq, 6 min dynamic scanning over heart). Then, a target activity of 2 MBq/kg [^68^Ga]Ga-ATH001 with a total protein mass <100 µg, conforming to microdosing regulations, was administered intravenously, followed by cardiac imaging using first a 15 min dynamic scan immediately after tracer administration, followed by a delayed 20 min static PET acquisition, performed 80 min post-injection. The simultaneous MRI protocol included late gadolinium enhancement (LGE) imaging for assessment of localization and extent of the myocardial infarct.

Image visualization of [^68^Ga]Ga-ATH001 was conducted using Carimas 2.10 software (Turku PET Center). PET data were normalized to Standardized Uptake Values (SUVs), normalized for injected radioactivity and patient body weight. Myocardial perfusion parametric images of ^15^O-H_2_O were generated in aQuant software (v. 1.2.0.54, Medtrace, Hørsholm, Denmark) [38].

## 5. Conclusions

In conclusion, we report the successful development, GMP-compliant production, and translational validation of [^68^Ga]Ga-ATH001, a novel Affibody-based PET radiopharmaceutical targeting PDGFRβ for imaging fibrotic remodeling in cardiovascular disease. The tracer production demonstrated high radiochemical purity, stability, and reproducibility using a fully automated synthesis process, which was optimized for clinical application. This established production process, combined with the use of commercially available synthesis platform, cassettes, and software, facilitates straightforward transfer to other medical production sites, thereby promoting broader implementation and supporting multicenter clinical trials.

Preclinical validation confirmed the tracer specific binding to PDGFRβ in fibrotic human myocardial tissue and mouse infarct model. Preliminary analysis of clinical imaging in STEMI patients further demonstrated focal tracer accumulation in infarcted myocardium, corresponding to areas of structural remodeling and perfusion deficits as well as demonstrating safety at microdosing levels.

These findings establish strong proof-of-concept for [^68^Ga]Ga-ATH001 as a clinically feasible molecular imaging agent for assessing active cardiac fibrosis. By enabling non-invasive visualization of fibrogenic activity, the tracer has the potential to guide personalized post-infarction care, evaluate response to anti-fibrotic therapies, and improve risk stratification in cardiac patients. Moreover, the production methodology presented here fulfills current technical and regulatory standards and is well-positioned to support global clinical adoption of [^68^Ga]Ga-ATH001.

## Figures and Tables

**Table 1:** Summary of the product specifications and validation results for three consecutive validation productions of [^68^Ga]Ga-ATH001.

Test	Acceptance Criteria	1	2	3
Radiochemical purity	≥91%; no unknown impurity corresponds to >5%	97.6	98.0	97.0
Chemical purity	Absence of unknown UV signals	>99	>99	>99
pH	4–8	7	7	7
Radioactivity concentration	5–100 MBq/mL	66	59	69
Radioactivity	100–800 MBq	596	508	562
Color	Colorless	Passed	Passed	Passed
Volume	8–10 mL	8.6	8.66	8.11
Radionuclidic purity	>99.9%	>99.9999	>99.9999	>99.9999
^68^Ge breakthrough	<0.001%	0.000005	0.000006	0.000004
Sterility test	sterile	sterile	sterile	sterile
Endotoxins test	<3 EU/mL	<0.25	<0.25	<0.25
Stability	RCP ≥ 91% within 120 min	96.1	96.9	97.4
Ethanol	<10%	2.5	2.2	2.1
Sterile filter integrity	≥1 bar, pressure hold test	Passed	Passed	Passed

**Table 2:** Summary of the UV-Radio-HPLC quality control method validation tests and results.

Description	Acceptance Criteria	Result
Specificity—UV detector (Relative standard deviation)	≤5%	0.5 (N = 8)
Linearity—UV detector (Pearson correlation coefficient, R^2^)	>0.99%	0.9999
Precision—UV detector (Relative standard deviation)	≤5%	1.38 (N = 6)
Specificity—Radio detector (Relative standard deviation)	≤5%	0.6 (N = 8)
Column recovery (free ^68^Ga)	≥95%	>99
Column recovery ([^68^Ga]Ga-ATHF)	≥95%	>99
Stability (during 2 h)	>91%	>96

**Table 3:** Tests and methods used for the quality control of each batch of [^68^Ga]Ga-ATHF.

Test	Acceptance Criteria	Method
Identity	Retention time corresponds to ATH001	UV-HPLC
Radiochemical purity	>91%, no unknown impurity corresponding to >5%	Radio-RP-HPLC
Chemical purity and peptide concentration	Absence of new unknown peaks in the UV-trace.	UV-RP-HPLC
Radioactivity	100–800 MBq	Ionization chamber
Specific radioactivity	5–50 MBq/nmol	Calculated
pH	4–8	pH indicator strip
Volume, [mL]	8–10	Scale
Color	Colorless	Visual inspection
Sterile filter integrity	Integrity test passed	Pressure hold test in-line

## Data Availability

The original contributions presented in this study are included in the article. Further inquiries can be directed to the corresponding author.

## Abbreviations

The following abbreviations are used in this manuscript:

GMP	Good Manufacturing Practice
GRPP	Good Radiopharmaceutical Practice (GRPP)
PET	Positron emission tomography
MRI	Magnetic resonance imaging
PDGFRβ	Platelet-derived growth factor receptor beta
STEMI	ST-elevation myocardial infarction
PCI	percutaneous coronary intervention
HPLC	High pressure liquid chromatography
